# Exploiting Integrin-αVβ3 to Enhance Radiotherapy Efficacy in Medulloblastoma via Ferroptosis

**DOI:** 10.3390/curroncol31110545

**Published:** 2024-11-20

**Authors:** Célia Gotorbe, Fabien Segui, William Echavidre, Jérôme Durivault, Thays Blanchard, Valérie Vial, Marina Pagnuzzi-Boncompagni, Rémy Villeneuve, Régis Amblard, Nicolas Garnier, Cécile Ortholan, Benjamin Serrano, Vincent Picco, Jacques Pouysségur, Milica Vucetic, Christopher Montemagno

**Affiliations:** 1 Biomedical Department, Centre Scientifique de Monaco, 98000 Monaco, Monaco; cgotorbe@centrescientifique.mc (C.G.); fsegui@centrescientifique.mc (F.S.); wechavidre@centrescientifique.mc (W.E.); jdurivault@centrescientifique.mc (J.D.); thaysblanchard98@gmail.com (T.B.); vial@centrescientifique.mc (V.V.); mpagnuzzi@centrescientifique.mc (M.P.-B.); vpicco@centrescientifique.mc (V.P.); jacques.pouyssegur@univ-cotedazur.fr (J.P.); 2 Radiophysics Department, Princess Grace Hospital, 98000 Monaco, Monaco; remy.villeneuve@chpg.mc (R.V.); regis.amblard@chpg.mc (R.A.); nicolas.garnier@chpg.mc (N.G.); benjamin.serrano@chpg.mc (B.S.); 3 Radiotherapy Department, Princess Grace Hospital, 98000 Monaco, Monaco; cecile.ortholan@chpg.mc; 4 CNRS, INSERM, Centre A. Lacassagne, Institute for Research on Cancer & Aging (IRCAN), University Côte d’Azur, 06107 Nice, France

**Keywords:** integrin-αvβ3, medulloblastoma, cilengitide, radiotherapy, ferroptosis

## Abstract

Medulloblastoma, a malignant pediatric brain tumor, has a poor prognosis upon relapse, highlighting a critical clinical need. Our previous research linked medulloblastoma cell radioresistance to integrin-αvβ3 expression. β3-depleted (β3_KO) medulloblastoma cells exhibit lipid hydroxyperoxide accumulation after radiotherapy, indicating ferroptosis, a regulated cell death induced by ROS and inhibited by antioxidants such as cysteine, glutathione (GSH), and glutathione peroxidase 4 (GPx4). However, the link between αvβ3 expression, ferroptosis inhibition, and sensitivity to radiotherapy remains unclear. We showed that irradiated β3_KO medulloblastoma cells primarily die by ferroptosis, with β3-subunit expression correlating with radiotherapy sensitivity and anti-ferroptotic protein levels. Our findings suggest that integrin-αvβ3 signaling boosts oxidative stress resilience via mTORC1. Thus, targeting integrin-αvβ3 could enhance radiotherapy efficacy in medulloblastoma by inducing ferroptotic cell death.

## 1. Introduction

Medulloblastoma (MB) is one of the most common malignant brain tumors in children, accounting for 20% of pediatric central nervous system (CNS) tumors [1]. Since 2016, the World Health Organization (WHO) has classified MB as a grade IV tumor based on its malignancy and treatment challenges—an unchanged classification [2,3]. The standard treatment for MB involves surgical resection followed by craniospinal irradiation and adjuvant chemotherapy [4,5]. While these treatments have significantly improved patient survival rates, they are associated with severe side effects, including neurological deficits and neurocognitive impairments [6]. Additionally, relapse occurs in about 30% of cases, leading to a poor prognosis [7,8,9]. Therefore, improving current therapies is critical, especially considering the young age of the affected patients.

Radiotherapy is a cornerstone of treatment, yet the recurrence observed in patients highlights the radioresistance of MB. An effective therapeutic strategy to address this challenge could involve enhancing the cytotoxic effect of radiotherapy on tumor cells by targeting key factors involved in radioresistance [10,11,12]. Our previous study identified integrin-αvβ3 as a crucial player in MB’s radioresistance [13]. Integrins are a superfamily of adhesion proteins involved in cell-extracellular matrix (ECM) interactions, consisting of 18 α-subunits and eight β-subunits forming at least 24 different receptors [14]. Integrin interactions with ECM components trigger intracellular signaling pathways that regulate cell growth and survival under physiological conditions. However, dysregulated integrin expression in various cancers, including brain tumors, leads to enhanced signaling that promotes uncontrolled proliferation and aberrant angiogenesis [15,16]. Among integrins, integrin-αvβ3 is highly expressed and crucial for the invasiveness of brain tumors. Its role has been extensively studied in glioblastoma, where its expression correlates with tumor grade, promotes angiogenesis, and facilitates cancer cell adhesion to the ECM and dissemination throughout the brain [16]. Similarly, our research demonstrated that integrin-αvβ3 is significantly expressed in a subpopulation of MB patients, and β3-subunit deletion impairs the tumorigenic capacities of MB. Moreover, integrin-αvβ3 was associated with the radioresistance of MB cells. Notably, we observed that β3-depleted cells exhibited a distinct bubbling phenotype characteristic of ferroptosis upon radiation exposure. Ferroptosis is a regulated, ROS- and iron-dependent form of cell death involving oxidative damage to plasma membrane lipids [17]. The canonical anti-ferroptotic pathway involves the cystine transporter (xCT), glutathione (GSH), and glutathione peroxidase 4 (GPX4), which detoxify lipid hydroperoxides. GPX4, a key player in this pathway, uses GSH to reduce lipid hydroperoxides to less toxic alcohol derivatives, with its activity dependent on cysteine import via the xCT transporter [18,19,20]. However, the relationship between radiotherapy-induced ferroptosis and integrin-αvβ3 and the connection between MB and ferroptosis has not yet been explored. In this study, we aimed to assess the role of integrin-αvβ3 in this process. First, we confirmed that ferroptosis is the predominant cell death pathway in β3-depleted MB cells following radiotherapy. Our analysis revealed that the increased sensitivity of these cells to radiation-induced ferroptosis is due to lower levels of antioxidant defenses, particularly GPX4 protein content, compared to their WT counterparts. The basal GPX4 content was lower in β3-depleted cells and remained low even after irradiation. We hypothesized that integrin-αvβ3 signaling controls GPX4 protein synthesis via the mTORC1 pathway. Our data were confirmed with the use of cilengitide, a pharmacological inhibitor of integrin-αvβ3 that enhances radiotherapy-induced ferroptosis. Therefore, integrin-αvβ3 is a promising therapeutic target for MB as an antitumoral agent and a sensitizer to ROS-inducing treatments. To the best of our knowledge, this is the first study that demonstrates the impact of integrin-αvβ3 in the mitigation of radiotherapy-induced ferroptosis.

## 2. Materials and Methods

### 2.1. Chemicals

Different chemicals were used in this study; cilengitide (M9173, Abmole, Houston, TX, USA), MG138 (474790, Sigma Aldrich, St. Louis, MO, USA), ferrostatin-1 (SML0583, Sigma Aldrich), necrostatin-1 (ab141053, Abcam, Cambridge, UK), Q-VD-OPH (T0282, TargetMol, Boston, MA, USA), erastin (E7781, Sigma Aldrich), RSL3 (SML2234, Sigma Aldrich), Torin-1 (S2827, Selleck Chemicals, Houston, TX, USA), and cycloheximide (2112S, CST, Danvers, MA, USA).

### 2.2. Cell Lines and Culture Conditions

Human MB cell lines were used in this study. The DAOY cell line (catalog number: HTB-186) was sourced from the American Type Cancer Collection (ATCC, Manassas, VA, USA). The HD-MB03 cell line was acquired from the Deutsche Sammlung von Mikroorganismen und Zellkulturen (DMSZ, Braunschweig, Germany, catalog number: ACC 740). DAOY cells were cultured in Dulbecco’s modified eagle medium (DMEM), which was supplemented with 1 mM sodium pyruvate, 2 mM Glutamax, and 7.5% fetal bovine serum (FBS). For HD-MB03 cells, Roswell Park Memorial Institute medium with an additional 7.5% FBS was used. All cell cultures were maintained at 37 °C in a humidified atmosphere with 5% carbon dioxide (CO_2_).

### 2.3. Genetic Disruption of β3-Integrin and Lentiviral Transductions

Generation of β3-integrin-depleted DAOY and HD-MB03-β3 overexpressing cells were generated in our laboratory as previously described [13].

### 2.4. RT-qPCR

Real-time reverse transcription-quantitative polymerase chain reaction (RT-qPCR) analyses were performed using human MB cell lines. Total mRNAs were isolated using the Nucleospin RNA Kit from Macherey-Nagel (Macherey-Nagel, Düren, Germany). Complementary DNA (cDNA) synthesis was carried out with the Maxima First Strand cDNA Synthesis Kit for RT-qPCR with dsDNase from Thermo Fischer Scientific (Waltham, MA, USA). Quantitative PCR was then performed on an Applied Biosystems StepOnePlus System, using TB Green Premix Ex Ta (Tli RNase H Plus; Takara Bio, San Jose, CA, USA) reagents. Primer sequences included: GPX4_Forward 5′-GAGGCAAGACCGAAGTAAACTAC-3′; GPX4_Reverse 5′-CCGAACTGGTTACACGGG-3′. The relative expression levels were calculated by the ΔCt method, with normalization to the reference *36B4* gene.

### 2.5. Immunoblotting

Cells lysis was performed in 1.5× Laemmli buffer, and protein concentrations were quantified with the Pierce BCA Protein Assay from Thermo Fisher Scientific. Protein extracts (15 μg) were subjected to electrophoresis on either 10% or 12% sodium dodecyl-sulfate-polyacrylamide gels and subsequently transferred to polyvinylidene difluoride membranes (Merck Millipore, Burlington, MA, USA). The membranes were blocked with 2% milk in phosphate-buffered saline (PBS) and incubated with the following anti-human antibodies: rabbit anti-β3-integrin (1:1000; 13166, Cell Signaling Technology [CST], Danvers, MA, USA), rabbit anti-xCT (1:1000; 126915, CST), rabbit anti-GPX4 (1:1000; ab125066, Abcam), rabbit anti-p-4EBP1 (1:1000; 1344345, CST), rabbit anti-4EBP1 (1:1000; 9644, CST), rabbit anti-p-70S6K (1:1000; 9202S, CST), rabbit anti-p-RPS6 (1:1000; 2215S, CST), anti-p-paxillin (1:1000; 69363, CST), and rabbit anti-Cyclin D1 (1:1000, ab134175, Abcam). Actin (1:5000; MA5-15739, Thermo Fisher Scientific), Tubulin (1:1000; 2128, CST), and HSP90 (1:1000; MA1-10379, Thermo Scientific) were used as the protein loading control. Immunoreactive bands were detected with horseradish peroxidase-coupled anti-mouse or anti-rabbit antibodies (CST) using the ECL System (Merck Millipore, Burlington, MA, USA).

### 2.6. Irradiation Procedure

Cell irradiation on DAOY-derived and HD-MB03-derived cells was performed using a photon beam of 6 MV delivered by a Varian linear accelerator (Novalis TrueBeam STX (Varian, Palo Alto, CA, USA) using a calibrated irradiation field of 20 × 20 cm^2^. Single doses of 2 to 8 Gy were delivered at 6.5 Gy/min with a 2 cm surface bolus application to ensure dose uniformity at a depth of 100 cm source to tray distance. Non-irradiated cells were given a sham irradiation as controls.

### 2.7. Colony Formation Assay

DAOY-derived and HD-MB03-derived cells were seeded into 60 mm plates after exposure to radiation (0 to 8 Gy) with initial cell numbers of 2000 for DAOY and 4000 cells for HD-MB03-derived cells, respectively. Approximately 10–14 days after seeding, when visible colonies had formed, the cells were washed with phosphate-buffered saline (PBS) and stained with Giemsa solution for 30 min. After staining, the plates were rinsed and air-dried before counting the colonies. Survival fractions were calculated by comparing the colony counts to those of the control cells.

### 2.8. Flow Cytometry

A total of 10,000 events per sample were analyzed using a BD FACSMelody cytometer (Becton Dickinson, Franklin Lakes, NJ, USA), and data was processed using the FlowJo software version vX.0.7 (Ashland, OR, USA). Cells were cultured in 6-well plates at a density of 150,000 cells per well, in triplicate for each condition, and maintained at 37 °C in a 5% CO_2_ atmosphere with their respective media. Following this, cells were treated with cilengitide (1 μM) for four hours before exposure to a 2 Gy irradiation, with experiments performed at the indicated time after treatments. Cell death was assessed 48 h following irradiation (2 Gy) using propidium iodide (PI; Invitrogen, Waltham, MA, USA). Both floating and adherent cells were collected, centrifuged, and then resuspended in FACS buffer (PBS, 0.2% BSA, and 2 mM ethylenediaminetetraacetic acid), with 2 μg/mL of PI added just before analysis. Detection of lipid hydroperoxides: MB Cells were seeded in 60 mm diameter dishes and irradiated (2 Gy) 24 h after plating. Another 24 h later, the BODIPY 581/591 C11 dye (Molecular Probes, Eugene, OR, USA) was introduced to the media at a final concentration of 2 μmol/L, and the cells were incubated for 30 min at 37 °C in a 5% CO_2_ environment, protected from light. Following incubation, the cells were washed twice with PBS, detached using Accutase (Dutscher, Bernolsheim, France), and resuspended in FACS buffer. The modal scaling option was utilized for data representation, ensuring that each peak is normalized to its mode, corresponding to the percentage of the maximum number of cells within each specific bin.

### 2.9. Statistics

Data are expressed as mean ± standard error of the mean (SEM). Comparisons between groups were conducted using the non-parametric Mann–Whitney test for two groups or two-way analysis of variance (ANOVA) for more than two groups, with multiple comparisons adjusted using Sidak’s test. A *p*-value of less than 0.05 was considered statistically significant.

## 3. Results

### 3.1. Integrin-αvβ3 Promotes Resistance to IR-Induced Ferroptosis

**Figure 1:**
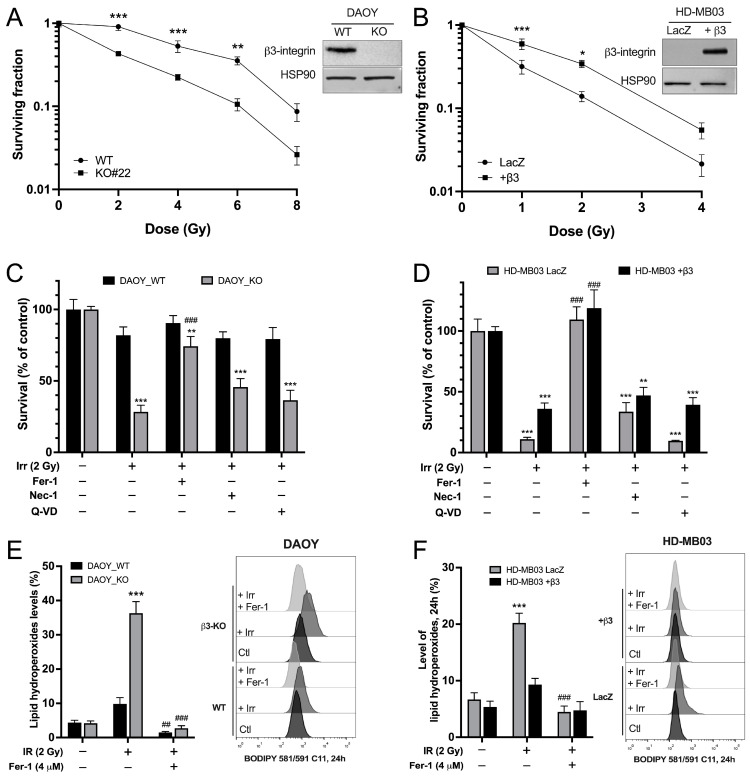
β3-depleted MB cells exhibit high sensitivity to IR-induced ferroptosis. (**A**,**B**) Representative western blots of β3-integrin expression in DAOY (control vs. β3_KO) and HD-MB03 (LacZ vs. β3+) cell lines. Survival curves of DAOY_WT vs. β3_KO cells (**A**) and HD-MB03-LacZ vs. HD-MB03-β3+ (**B**) after a single IR-indicated dose (Gy). Survival was determined by the percentage of unirradiated cells (Log10 scale). * *p* < 0.05, ** *p* < 0.01, *** *p* < 0.001 vs. corresponding controls. (**C**,**D**) Survival fraction of DAOY_WT and β3_KO (**C**) or HD-MB03_LacZ and +β3 (**D**) treated with 10 μM necrostatin-1 (Nec-1), 10 μM Q-VD-OPH, or 10 μM ferrostatin-1 (Fer-1) or DMSO (control) and exposed or not to a single dose (2 Gy) of IR. ** *p* < 0.01, *** *p* < 0.001 vs. corresponding control; ### *p* < 0.001 vs. corresponding IR group. (**E**,**F**) Lipid hydroperoxide content in DAOY (WT and β3_KO) and HD-MB03 (LacZ and β3+) cells 24 h after exposure to a 2 Gy IR in the presence or not of Fer-1 (4 μM) measured by C11-BODIPY 581/591 staining. *** *p* < 0.001 vs. corresponding control; ## *p* < 0.01; ### *p* < 0.001 vs. corresponding IR group. Results are expressed in mean ± SEM. Results are expressed as mean ± SEM. Data points represent three independent biological experiments, with each experiment shown as individual dots. Original uncropped western blot membrane figures can be found in Supplementary File S1.

Two human MB cell lines (DAOY and HD-MB03) were used to investigate the role of integrin-αvβ3 in the radiosensitivity of MB. The expression of β3-integrin was restricted to the DAOY cell line. Genetic depletion or overexpression of β3-subunit was performed respectively on the DAOY and HD-MB03 cells (Figure 1A,B).

HD-MB03_LacZ was used as a non-relevant protein expression control for HD-MB03_β3+ cells. The generation of these MB-derived cell lines prompted us to analyze the impact of this protein on the radioresistance of MB. Our data clearly showed a decrease in cell survival upon IR in a dose-dependent manner in both DAOY_WT and KO cells. However, integrin-β3_KO cells were significantly more sensitive to IR than WT cells at every investigated dose (Figure 1A). Similar results were found in HD-MB03_LacZ in comparison to HD-MB03_β3+ cells, with a significantly higher survival rate for HD-MB03-β3+ at 1 and 2 Gy (Figure 1B).

Next, the type of cell death induced by IR in MB cell lines was investigated using inhibitors of necroptosis, necrostatin-1 (Nec-1); apoptosis, quinolyl-valyl-O-methylaspartyl-[-2,6-difluorophenoxy]-methylketone (Q-VD) and ferroptosis, ferrostatin-1 (Fer-1) (Figure 1C,D). As previously observed, a 2 Gy irradiation reduced cell survival to a greater extent in DAOY_KO cells than in their WT counterparts (81.9% ± 6.9% vs. 28.3% ± 4.6% of cell survival, Figure 1C). Consistently, HD-MB03-β3+ cells displayed a better survival than the control HDMB_LacZ cells (Figure 1D). Pre-treatment of MB cells with Fer-1 before IR was found to rescue IR-induced cell death in all investigated MB-derived cells (Figure 1C,D). No significant rescue was observed with necroptosis or apoptosis inhibitors, highlighting that cells mainly die of ferroptosis when exposed to IR.

As a major hallmark of ferroptosis, lipid peroxidation was investigated in irradiated MB cell lines. C11-BODIPY staining revealed that 2 Gy IR induced lipid peroxidation exclusively in integrin-αvβ3 negative MB cells (Figure 1E,F). Indeed, following irradiation, lipid hydroperoxides content was four times higher in DAOY_KO than in DAOY_WT cells (36.3% ± 3.4% vs. 9.8% ± 1.8%) and twice as high in HD-MB03_LacZ compared to HD-MB03_β3+ cells (20.2% ± 1.7% vs. 9.3% ± 1.1%, respectively) (Figure 1E,F). Cell death and lipid peroxide accumulation in DAOY_KO and HD-MB03_LacZ 24 h post-irradiation were completely rescued by Fer-1 (Figure 1E,F and Figure S1A,B). Together, our results demonstrated the importance of β3-integrin in responding to IR-induced ferroptosis.

Given the vulnerability of DAOY_KO cells to ferroptosis, we hypothesized that targeting GPX4 or xCT could synergize with the depletion of integrin-αvβ3. However, genetic depletion of xCT or GPX4 induces extensive cell death, complicating synergy studies. Pharmacological inhibition allows precise dosing to minimize cell death, enabling the exploration of combinatorial effects with β3 inhibition. However, inhibition of GPX4 with RSL3 or xCT with erastin yielded a similar effect on both DAOY_WT and DAOY_KO cells (Figure S2A,B). Protein expression analysis in both DAOY-derived cells showed an increased level of xCT upon RSL3 treatment (Figure S2C). Such compensatory mechanisms could account for the lack of cumulative effect of β3-depletion and GPX4 or xCT inhibition on the clonogenic capacities of DAOY cells.

### 3.2. Integrin-αvβ3 Regulates Antioxidant Protein Expression

**Figure 2:**
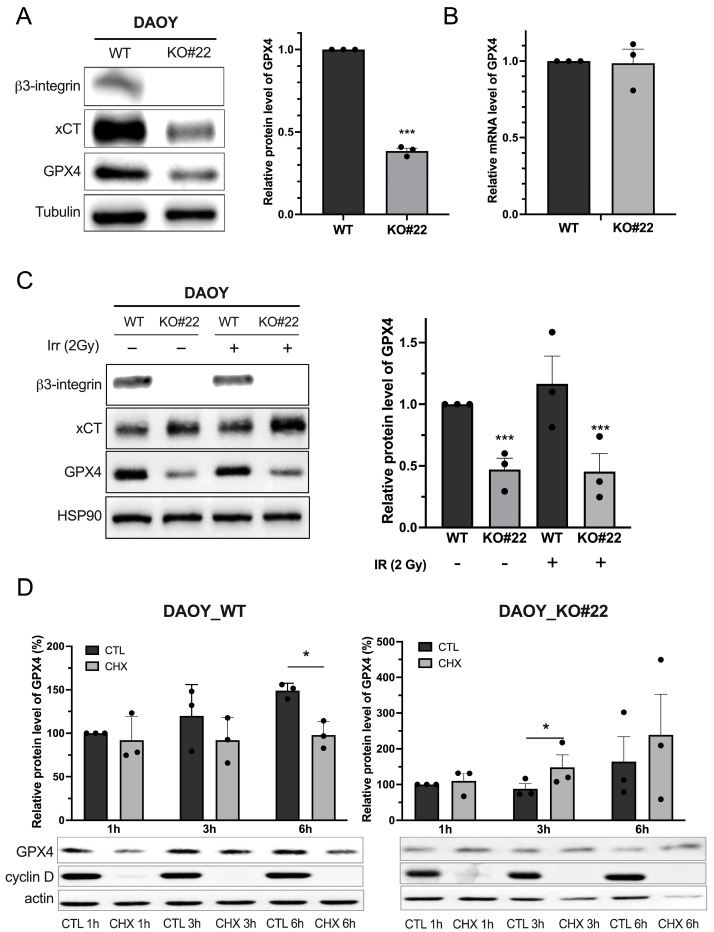
β3-depleted MB cells have compromised anti-ferroptotic defense. (**A**) Protein contents of the major anti-ferroptotic players’ xCT and GPX4 were analyzed by Western blot in DAOY (WT and β3_KO). Relative GPX4 protein levels are presented as mean ± SEM. ***, *p* < 0.001 vs. control. (**B**) Gene expression of GPX4 in DAOY (WT and β3_KO) was analyzed by RT-qPCR. Results are expressed in the function of 36B4 gene expression. (**C**) Protein content of xCT and GPX4 in DAOY (WT and β3_KO) was investigated 24 h after a single dose (2 Gy) of IR. Quantification of GPX4 expression is represented on the right panel. ***, *p* < 0.001 vs. DAOY_WT. (**D**) Cycloheximide (CHX) chase assay for the half-life of GPX4 performed in DAOY_WT and DAOY β3_KO cells. Cells were treated with CHX (100 μg/mL) for the indicated hours, and Western blotting was performed. The level of remaining GPX4 at different time points was quantified as the percentage of the initial GPX4 level. *, *p* < 0.05 vs. corresponding control. Results are expressed in mean ± SEM. Results are expressed as mean ± SEM. Data points represent three independent biological experiments, with each experiment shown as individual dots. Original uncropped western blot membrane figures can be found in Supplementary File S1.

The crucial role of integrin-αvβ3 in the resistance of IR-induced ferroptosis prompted us to investigate the underlying molecular mechanisms. The expression of the major components of the antioxidant axis xCT and GPX4 was first assessed in MB-derived cell lines (Figure 2). Levels of both proteins were found to be lower in DAOY_KO and HD-MB03_LacZ cells (Figure 2A and Figure S3).

Specifically, GPX4 protein, a major actor involved in removing lipid hydroperoxides in the plasma membrane, was approximately 70% lower in DAOY_KO cells in comparison to WT ones (Figure 2A). Similarly, HD-MB03_ β3+ displayed a 50% increase in GPX4 expression compared to HD-MB03_LacZ (Figure S3). This significant difference persisted post-irradiation, highlighting the strong vulnerability of DAOY_KO cells to this stress (Figure 2C). On the contrary, xCT expression, a major component of the antioxidative defense, significantly increased following irradiation in DAOY_WT and DAOY_KO cells (Figure 2C).

To investigate the influence of integrin-αvβ3 on GPX4 expression, we examined whether this regulation occurs at the transcriptional level. Manipulation of β3 expression levels in both HD-MB03 and DAOY cell lines did not alter GPX4 expression (Figure 2B and Figure S3B). This suggests that β3 integrin is not involved in regulating GPX4 mRNA levels. Additionally, we assessed the effect of integrin-αvβ3 on GPX4 protein stability using a cycloheximide (CHX) chase assay on DAOY-derived cells (Figure 2D). No differences in GPX4 stability were observed under the tested conditions. To further exclude β3 integrin-dependent regulation of GPX4 stability, we investigated proteasome-mediated degradation (Figure S4). Our results indicate that integrin-αvβ3 does not regulate GPX4 protein stability.

### 3.3. Integrin-αvβ3 Regulates GPX4 Expression by Modulating mTORC1 Axis

**Figure 3:**
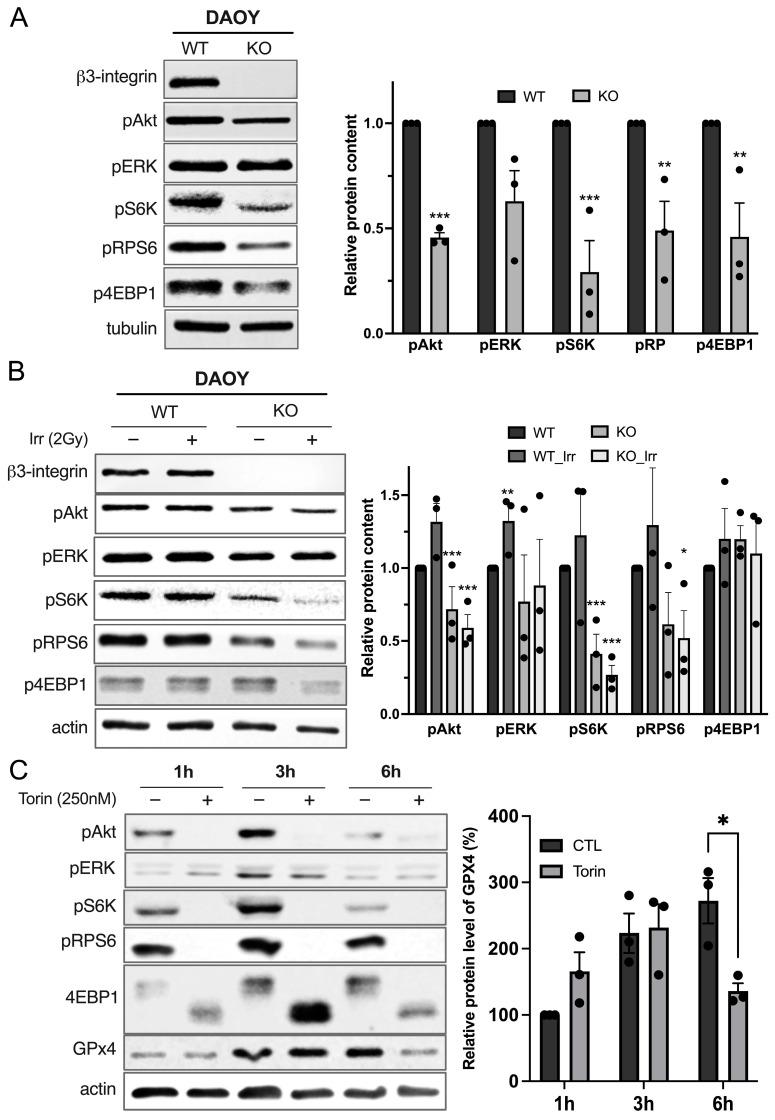
Integrin-αvβ3 controls GPX4 protein level through its action on mTORC1/4EBP1 axis. (**A**) Protein levels of αvβ3 signaling, including different cellular targets of mTORC1 complex: 4EBP1, 70S6K, and S6RP, in their phosphorylated form, were measured by Western blot in DAOY. Protein expression in western blots was quantified by densitometry, and the results are expressed in comparison to DAOY_WT. **, *p* < 0.01; ***, *p* < 0.001 vs. DAOY_WT. (**B**) Protein levels of αvβ3 signaling were measured by Western blot in DAOY_WT and DAOY_β3_KO 24 h after a 2-Gy IR. Protein expression in western blots was quantified by densitometry, and the results are expressed in comparison to DAOY_WT. *, *p* < 0.05; **, *p* < 0.01; ***, *p* < 0.001 vs. DAOY_WT. (**C**) Protein levels of the indicated protein were measured by Western blot after treatment of DAOY_WT and DAOY_β3_KO after Torin treatment (250 nM, at the indicated time). Protein expression in western blots was quantified by densitometry, and the results are expressed in comparison to control untreated cells. *, *p* < 0.05; vs. Control. Results are expressed as mean ± SEM. Data points represent three independent biological experiments, each shown as individual dots. Original uncropped western blot membrane figures can be found in Supplementary File S1.

Given the pivotal role of integrins in cell growth and proliferation and given the fact that GPX4 expression level is not transcriptionally regulated by the integrin-αvβ3 nor through protein stability regulations, we postulated that the signaling from integrin-αvβ3 might globally induce protein translation. mTORC1 is a major cellular hub responsible for protein synthesis. Recent studies in the ferroptosis field have identified mTORC1 as a negative regulator of ferroptosis in cancer cells. Consistent with previous findings, DAOY_KO cells showed decreased PI3K/Akt and ERK signaling (Figure 3A). Additionally, a significant decrease in mTORC1 downstream phosphorylated targets, such as pS6K, pRPS6, and p4EBP1, was observed in DAOY_KO cells in comparison to DAOY_WT cells. Similar results were observed in HD-MB03-derived cells (Figure S5). This decrease was maintained upon irradiation (Figure 3B). To determine if decreased mTORC1 activity affects GPX4 protein levels, the effect of Torin1, a potent and selective ATP-competitive mTOR inhibitor, was evaluated on DAOY cells (Figure 3C). A decrease in phosphorylated-mTORC1 targets was observed at every investigated time point, and GPX4 expression also decreased following a six-hour Torin-1 treatment. Together, our results suggest that mTORC1 pathway activation induces the synthesis of GPX4 (Figure S6).

### 3.4. Cilengitide Mimics Radiosensitivity Induced by β3-Depletion

To mimic β3-depletion, we explored the pharmacological impact of cilengitide, an RGD-derived compound, on IR-induced ferroptosis. Pre-treatment of DAOY cells with cilengitide induced a 2-fold increase in cell death after IR compared to the IR-alone group (25% ± 1.9% vs. 11.8% ± 1.4%, *p* < 0.001) (Figure S7A). Consistently, an increase of lipid hydroperoxides accumulation was found in cilengitide pre-treated cells after irradiation (42.9% ± 8.1% vs. 11.7% ± 0.9%, *p* < 0.01, Figure S6B). These effects were totally rescued with the use of Fer-1 (Figure S7A,B). Targeting integrin-αvβ3, therefore, appears to be an efficient way to increase the sensitivity of MB cells to IR-induced ferroptosis. We next used cilengitide to evaluate the regulation of the mTORC1 axis by integrin-αvβ3 signaling (Figure S7C). Cilengitide treatment decreased p-paxillin expression, a known downstream target of integrin-αvβ3, at four hours post-treatment. Disruption of integrin-αvβ3 signaling led to a significant reduction in p-70S6K, p-RPS6, and p-4EBP1 levels at the same time point. Additionally, a decrease in GPX4 protein content was observed in correlation with these results. Between eight and twenty-four hours of cilengitide treatment, the phosphorylation levels of all investigated proteins and GPX4 protein content were restored (Figure S7C). These results indicate that pharmacological inhibition of integrin-αvβ3 phenocopies the genetic deletion of its β3-subunit.

## 4. Discussion

Craniospinal irradiation (CSI) is the cornerstone of MB treatment [5]. Over the past few decades, significant efforts have been made to enhance CSI efficacy, including its combination with chemotherapy. However, relapse and resistance to these therapies underscore the need for new therapeutic strategies. Targeting key pathways of MB resistance, such as those involved in DNA damage response and telomerase activity, represents a promising approach [21,22,23]. Among alternative targets to overcome tumor radioresistance, integrins have been extensively studied across various cancers and present an attractive option [24]. In line with studies on adult tumors, our previous work demonstrated that integrin-αvβ3 promotes tumorigenesis and could be a potent therapeutic target for radiosensitization of MB cells [25]. The up-regulation of integrin-αvβ3 in radioresistant cell lines and their dependency on this complex could open new avenues for this issue.

Healthy and cancerous mammalian tissues are composed of about 70 to 80% water [26]. Therefore, the primary effect of IR imposed on cancerous lesions is water radiolysis, leading to ROS formation and subsequent oxidative damage to nucleic acids, proteins, and membrane lipids [27,28,29]. Collectively, these effects can induce cell cycle arrest, senescence, and apoptosis [30]. By means of lipid peroxidation measurement and pharmacological rescue, we showed for the first time that radiotherapy primarily induces ferroptosis in β3-negative MB cells. Since 2012, ferroptosis has been recognized as a novel-ROS-dependent type of cell death, characterized by the accumulation and uncontrolled diffusion of lipid hydroperoxides within the plasma membrane [17]. Despite significant oxidative damage to membrane lipids caused by IR-induced water radiolysis, the role of ferroptosis in radiotherapy was not reported until recently [31]. The first evidence showing the involvement of ferroptosis in response to radiotherapy was recently reported in mouse models of ovarian cancer [32]. Subsequent studies on various tumor types have further confirmed the link between radiotherapy and ferroptosis [33,34]. Consistently, we showed here for the first time that radiotherapy-induced ferroptosis in MB, with integrin-αvβ3 playing a pivotal role in counterbalancing this process. First, we demonstrated that β3-depleted cells are more sensitive to radiotherapy compared to β3-proficient cells. Our data suggest that this increased sensitivity to radiotherapy-induced ferroptosis arises, at least in part, from a compromised anti-ferroptotic antioxidant defense. Our analysis revealed a lower basal level of the canonical players of ferroptosis-preventing pathways, particularly GPX4, in β3-depleted DAOY and HD-MB03 cells, indicating a compromised internal anti-ferroptotic axis in these cells. Moreover, after radiotherapy, β3-depleted cells did not show an increase in GPX4 protein levels, in contrast to xCT, which was significantly upregulated upon radiotherapy in both wild-type and β3-deficient cells. These results suggest that oxidative stress induced by radiotherapy is sensed by both β3-proficient and β3-deficient cell lines, but the latter bears a higher burden of acute oxidative insult, likely due to an insufficient internal antioxidant response. Previous studies on integrin-dependent sensitivity to ferroptosis showed somewhat similar trends. Deletion of integrins such as αvβ3 or α6β4 increases cell sensitivity to ferroptosis, partly due to loss of xCT stability or increased synthesis of polyunsaturated fatty acids by the enzyme Acyl-CoA Synthetase Long-Chain Family Member 4 (ACSL4) [35,36]. Interestingly, in both studies, the deletion of integrins also led to a reduction in GPX4 protein levels, a point that was not addressed by the authors. Considering the absence of influence of β3-integrin on GPX4 mRNA levels and GPX4 protein stability (as seen in CHX chase experiments), we hypothesized that integrin-αvβ3 regulates its expression by influencing protein synthesis. We previously demonstrated that integrin-αvβ3 induces activation of Akt, a positive regulator of the major protein synthesis regulator, mTORC1. Consistent with these results, we showed that disruption of integrin-αvβ3 signaling decreased mTORC1 activity, as reflected by the reduced phosphorylation of S6K and 4EBP1. These findings align with data showing the impact of other integrins on mTORC1 activity [37,38]. To our knowledge, this is the first time that the AKT/mTORC1/4EBP1/GPX4 axis has been investigated downstream of the integrin-αvβ3 signaling.

The data presented here also address an important and still controversial question: the regulation of GPX4 expression. One frequently mentioned regulator of GPX4 is nuclear factor erythroid 2-related factor 2 (NRF2), a well-known sensor of the cellular redox state [39,40,41]. However, our previous studies suggest that this might not be the case. In xCT-depleted cells experiencing high oxidative stress and increased NRF2 signaling, the content of GPX4 remained markedly lower compared to their WT counterparts [42]. According to two recent studies on GPX4 regulation, a potential reason for this might be the suppressed activity of mTORC1 observed in cells upon cysteine starvation [43,44]. To investigate this issue, we treated the cells with Torin-1, a selective ATP-competitive mTORC1 inhibitor. Here, mTORC1 inhibition correlates with decreased GPX4 protein levels four hours after treatment. Torin-1 induced incomplete dephosphorylation of 70S6K and RPS6. However, a complete decrease of 4EBP1 phosphorylation, a “good quality” substrate of mTORC1, was found upon Torin-1 treatment. This result suggests that this branch of mTORC1 might be responsible for the protein synthesis of GPX4. 4EBP1 acts as a scavenger of eIF4E. When 4EBP1 is phosphorylated by mTORC1, eIF4E participates in recognizing the stabilizer CAP (m7GpppNm) present at the 5′ end of the mRNA, allowing the translation initiation of proteins such as GPX4. This aligns with Zhang et al.’s study, which showed that GPX4 is regulated by a Rag-mTORC1-4EBP1 axis. Interestingly, despite the proven efficacy of these inhibitors, GPX4 protein levels began to increase post-treatment of eight hours and were fully restored by 24 h, likely due to cellular adaptation to pharmacological mTORC1 inhibition (Figure S5).

We used cilengitide, an RGD-derived compound, to validate through a pharmacological approach the data generated by the genetic depletion of β3-integrin. Cilengitide has shown promise as a therapeutic compound due to its anti-tumor effects and ability to pass the blood-brain barrier [45,46,47]. We observed a decrease in GPX4 expression and mTORC1 activity starting four hours after cilengitide treatment. This finding aligns with the radiosensitizing effect of cilengitide. Moreover, our data underscore the necessity of precise timing for drug administration, considering that GPX4 levels and mTORC1 activity were restored from eight hours and further increased after 24 h, suggesting a compensatory mechanism like the one observed with torin-1. The transient nature of cilengitide’s effects on GPX4 and mTORC1 activities suggests that its therapeutic window is narrow, necessitating careful consideration of dosing schedules to sustain its anti-tumor effects. While the in vitro half-life of cilengitide is unknown, clinical studies report a plasma half-life of 3–5 h post-intravenous infusion [48]. In an orthotopic model of glioma, administering cilengitide shortly before radiotherapy was found to enhance tumor response, demonstrating the importance of treatment scheduling [48]. Additionally, further investigation into the compensatory mechanisms that restore GPX4 and mTORC1 activities post-treatment could reveal new targets for enhancing the efficacy of cilengitide in clinical settings.

In conclusion, our findings underscore the therapeutic potential of targeting integrin-αvβ3 and the mTORC1/GPX4 pathway to induce ferroptosis and overcome radioresistance in MB. To the best of our knowledge, this is the first study that demonstrated the impact of integrin-αvβ3 in radiotherapy-induced ferroptosis.

## Data Availability

All the datasets shown in the figures are available from the corresponding author upon reasonable request.

## Supplementary Materials

The following supporting information can be downloaded at: https://www.mdpi.com/article/10.3390/curroncol31110545/s1, Figure S1: β3-depleted MB cells are sensitive to IR-induced ferroptosis; Figure S2: β3-proficient and deficient DAOY cells show the same sensitivity to ferroptosis inducers; Figure S3: Overexpression of β3 in HD-MB03 increases GPX4 protein expression; Figure S4: Proteasome inhibition assay in DAOY-derived cells; Figure S5: Integrin-αvβ3 controls GPX4 protein level through its action on mTORC1/4EBP1 axis; Figure S6: Schematic representation of the hypothetic regulation of GPX4 protein synthesis by integrin-αvβ3 signalization; Figure S7: Pharmacological inhibition of integrin-αvβ3 by cilengitide phenocopies radiosensitivity of the genetic β3-deletion; File S1: Original uncropped western blot membrane figures.
