# Supplementary material for: Exploiting Integrin-αVβ3 to Enhance Radiotherapy Efficacy in Medulloblastoma via Ferroptosis
Source: Curr Oncol. 2024 Nov 20;31(11):7390–402. doi: 10.3390/curroncol31110545 (PMC11592711; doi:10.3390/curroncol31110545)
Supplement: Supplementary file 1 [file curroncol-31-00545-s001.zip › curroncol-3244536-supplementary.pdf]

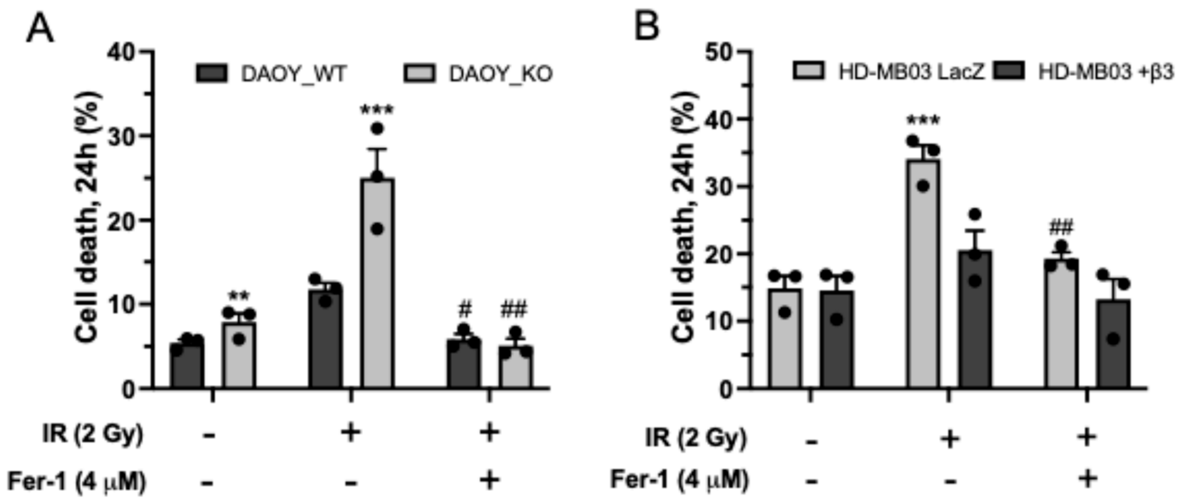

Figure S1. β3-depleted MB cells are sensitive to IR-induced ferroptosis. (A,B) Cell death in DAOY- (A) and HD-MB03- derived cells (B) was measured by PI incorporation (FACS) 24h after a 2-Gy IR, in presence or not of Fer-1. **$p<0.05$; ***$p<0.001$ vs corresponding control; # $p<0.05$; ## $p<0.01$ vs corresponding IR group. Results are expressed as mean ± SEM. Data points represent three independent biological experiments, with each experiment shown as individual dots.

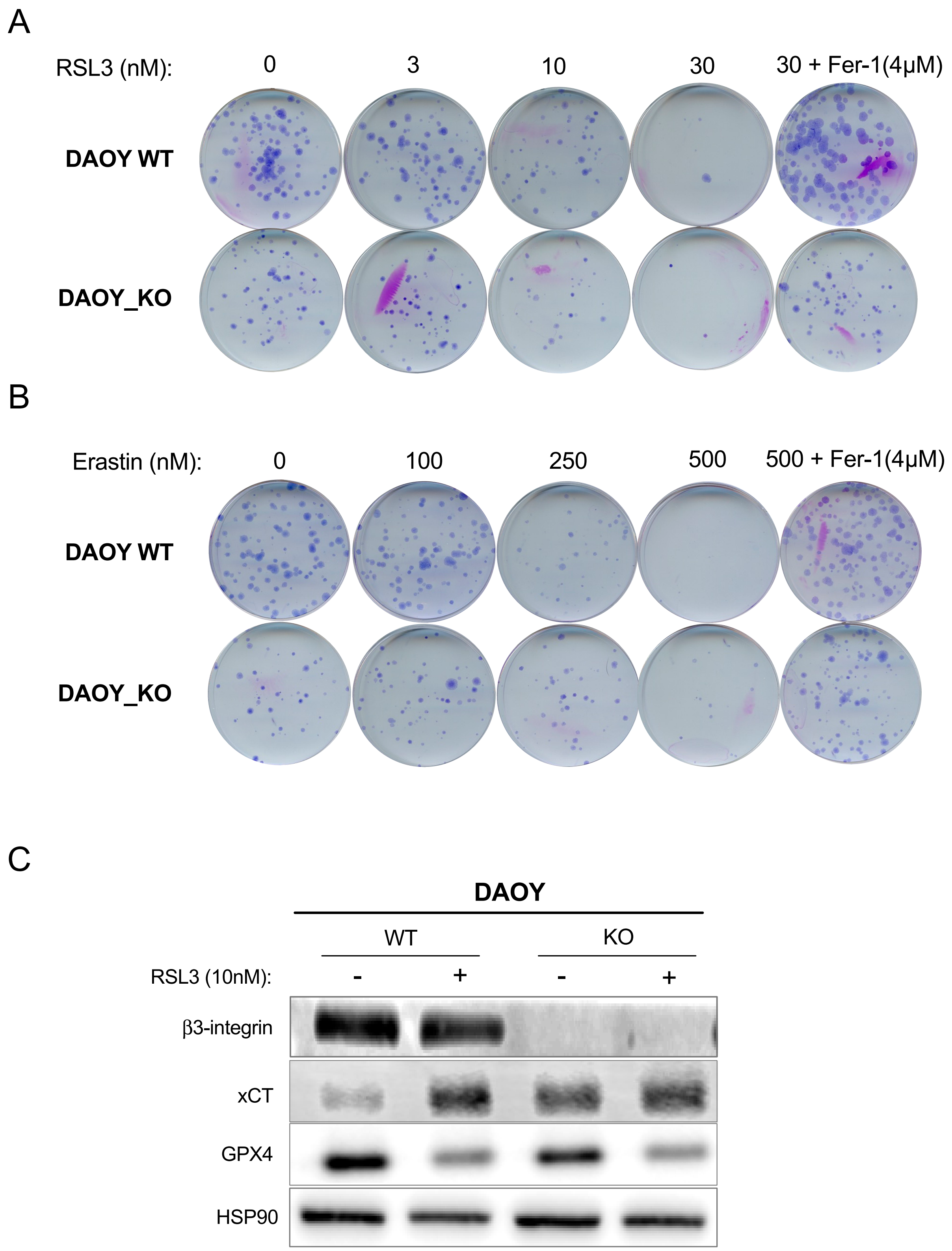

Figure S2. β3-proficient and deficient DAOY cells show same sensitivity to ferroptosis inducers. (A,B) DAOY WT and β3-KO cells were cultivated in DMEM media supplemented or not with increasing concentration of GPX4 inhibitor - RSL3 (0, 3, 10, 30 nM) (A) or xCT inhibitor - Erastin (0, 100, 250, 500 nM) (B), in the presence or not of Fer-1 (4μM). After 14 days colonies were colored for visualization using Giemsa. Representative images are shown. (C) Protein contents of xCT and GPX4 were analyzed

by Western blot in DAOY (WT and β3-KO) after 24h of RSL3 (10nM) treatment. Blots are representative of three independent experiments.

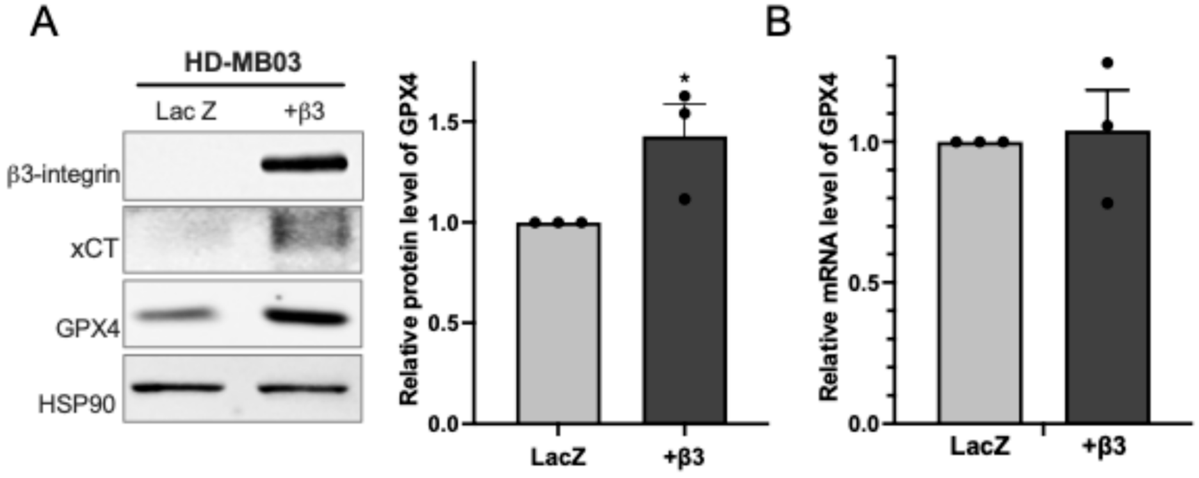

Figure S3. Overexpression of β3 in HD-MB03 increases GPX4 protein expression. (A) Protein contents of the major anti-ferroptotic players xCT and GPX4 were analyzed by Western blot in HD-MB03 (LacZ and β3+). Quantification of GPX4 expression is plotted on the right panel. * $p<0.05$ vs HD-MB03_LacZ. (B) mRNA expression of GPX4 in HD-MB03 (LacZ and β3+). Results are expressed as mean ± SEM. Data points represent three independent biological experiments, with each experiment shown as individual dots

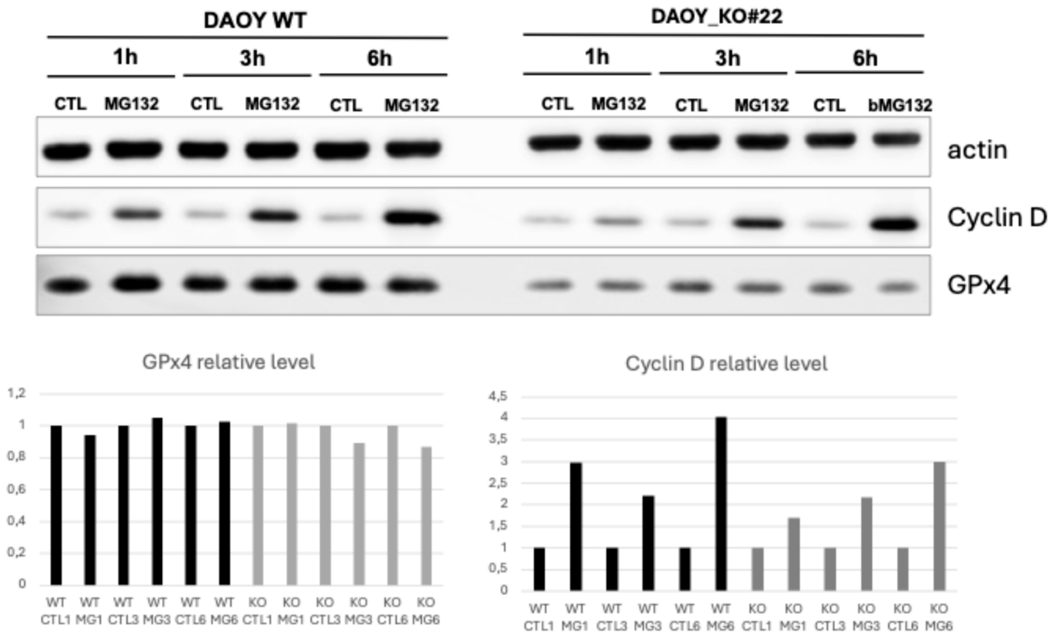

Figure S4. Proteasome inhibition assay in DAOY-derived cells. DAOY WT and β3-KO cells were treated with the proteasome inhibitor MG132 (10 µM, at the indicated time points) to assess the role of β3 in regulating GPX4 stability via the proteasomal degradation pathway. Protein levels of GPX4 and

Cyclin D (internal control) were measured by Western blot. Relative quantities of GPX4 and Cyclin D are shown at the bottom.

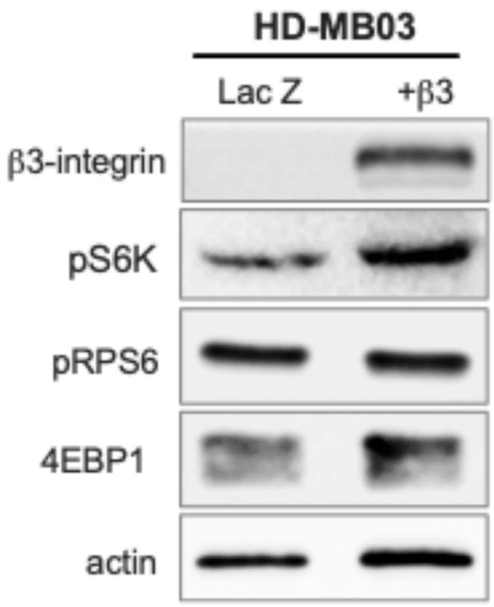

Figure S5. Integrin-αvβ3 controls GPX4 protein level through its action on mTORC1/4EBP1 axis. Protein levels of different cellular targets of mTORC1 complex: 4EBP1, and 70S6K and S6RP in their phosphorylated form were measured by Western blot in HD-MB03-derived cells.

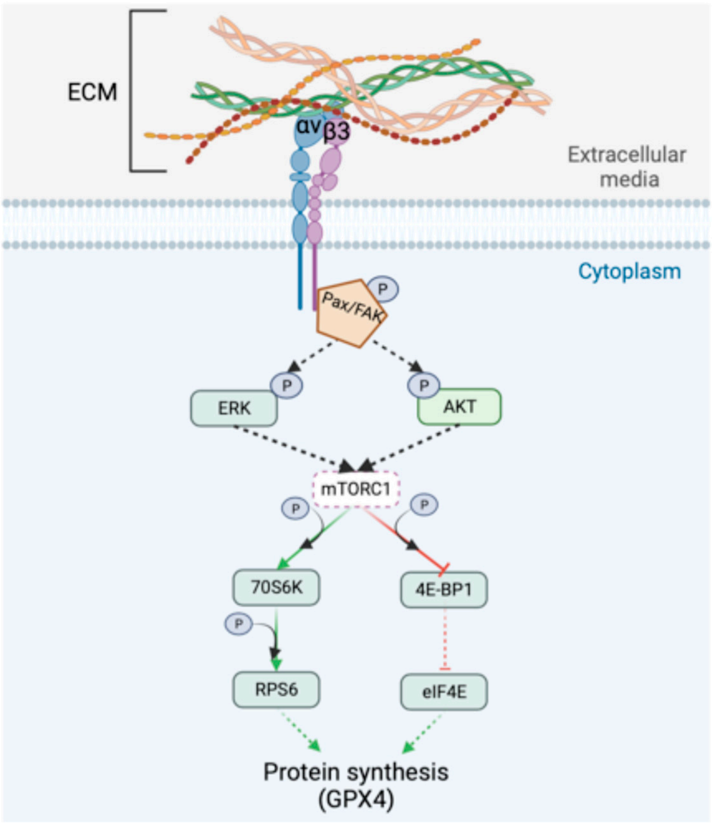

Figure S6. Schematic representation of the hypothetic regulation of GPX4 protein synthesis by integrin-αvβ3 signalization: The interaction of integrin-αvβ3 with its substrates of the ECM induces activation of cellular kinases such as ERK and AKT through the action of focal adhesion kinase (FAK)/paxillin (Pax). These kinases are well-known positive regulators of the mTORC1, which from its side induces phosphorylation and inhibition of 4EBP1, allowing the release of eiF4E and initiation of translation and protein synthesis of GPX4.

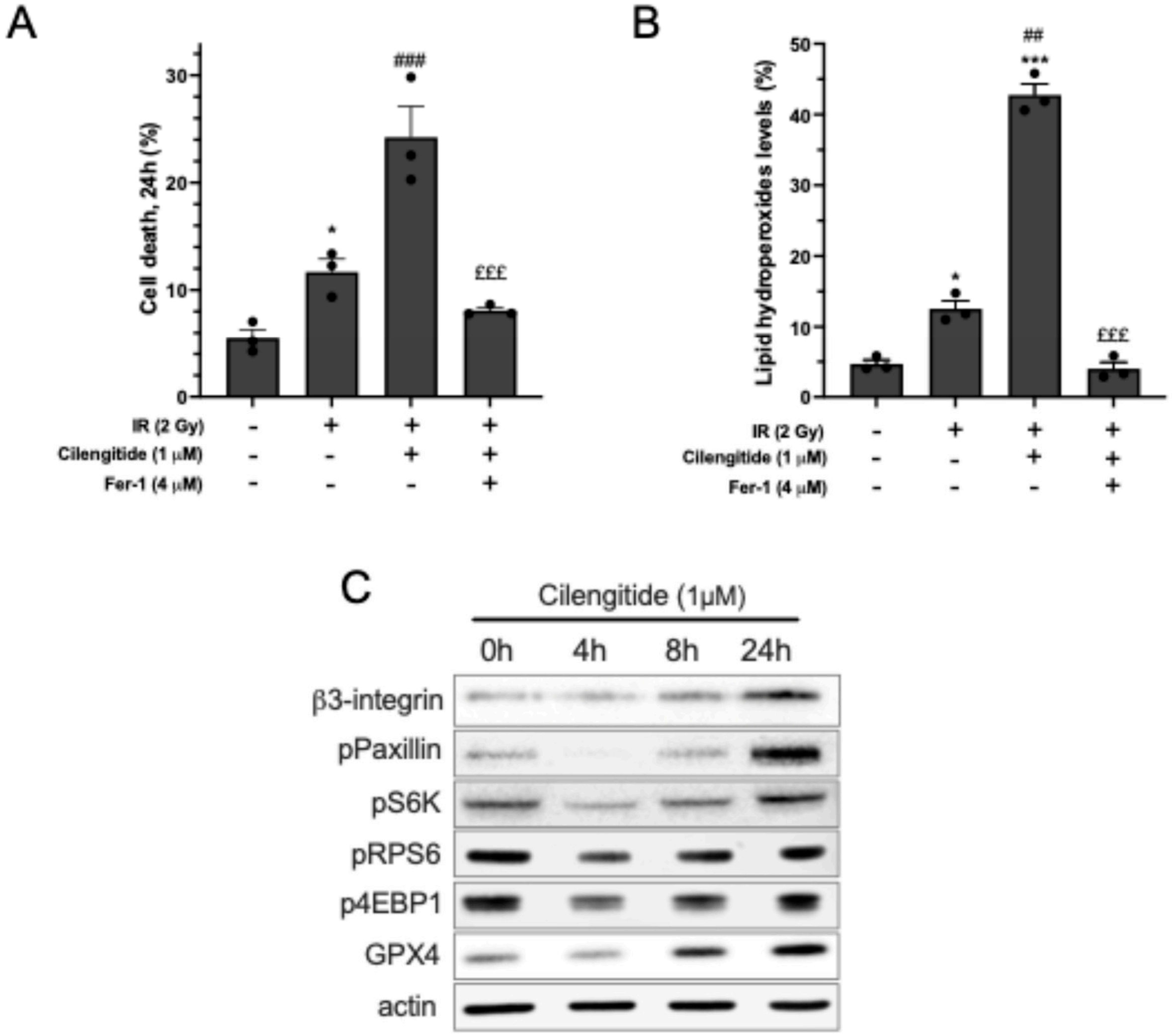

Figure S7. Pharmacological inhibition of integrin-αvβ3 by cilengitide phenocopies radiosensitivity of the genetic β3-deletion. (A) Cell death and (B) lipid hydroperoxides content assessed in DAOY_WT cells. Cells were pre-treated for 4h with 1 μM cilengitide, and cell death was measured 24h post single IR dose (2Gy) in the presence or not of 4 μM Fer-1. Results are expressed as mean ± SEM. Data points

represent three independent biological experiments, with each experiment shown as individual dots. *, $p<0.05$; ***, $p<0.001$ vs non-irradiated control group. ##$p<0.01$ ###$p<0.001$ vs IR control group and £££, $p<0.001$ vs IR treated group. (C) Protein levels of different mTORC1 downstream targets (p4EBP1, p70S6K and pRPS6) as well as GPX4 protein content, were analyzed by Western blot in DAOY WT following cilengitide treatment (1μM) at indicated time points. Blots are representative of three independent experiments.

File S1. Original uncropped western blot membrane figures

Figure 1A

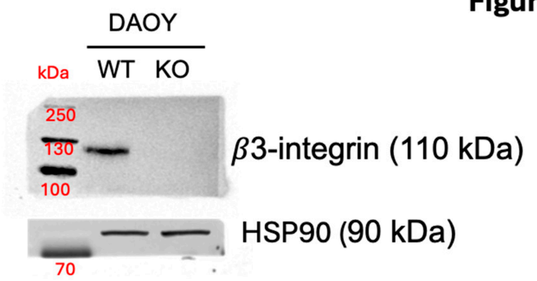

Figure 1B

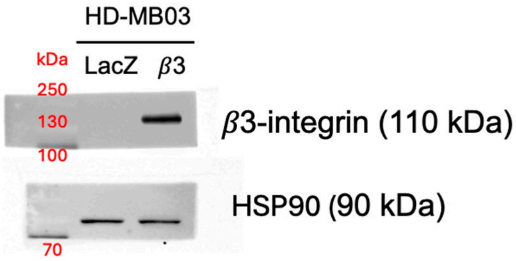

Figure 2A

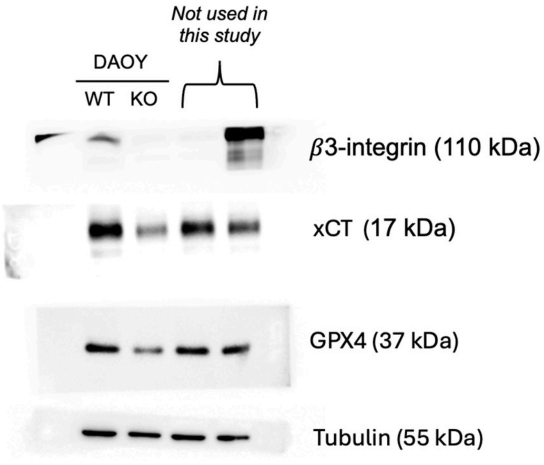

Figure 2C

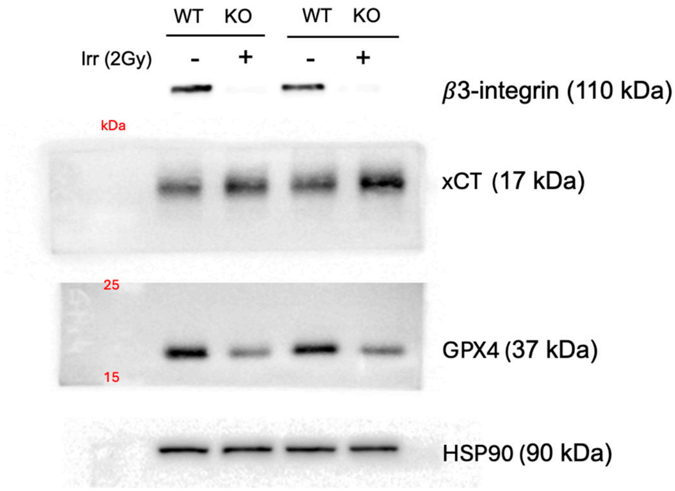

Figure 2D

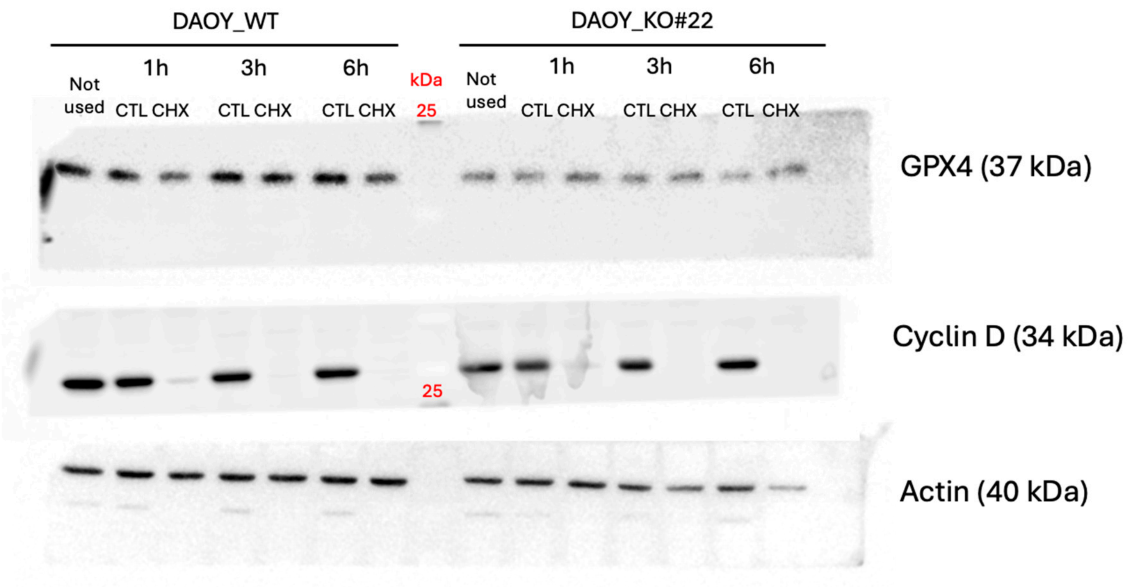

Figure 3A

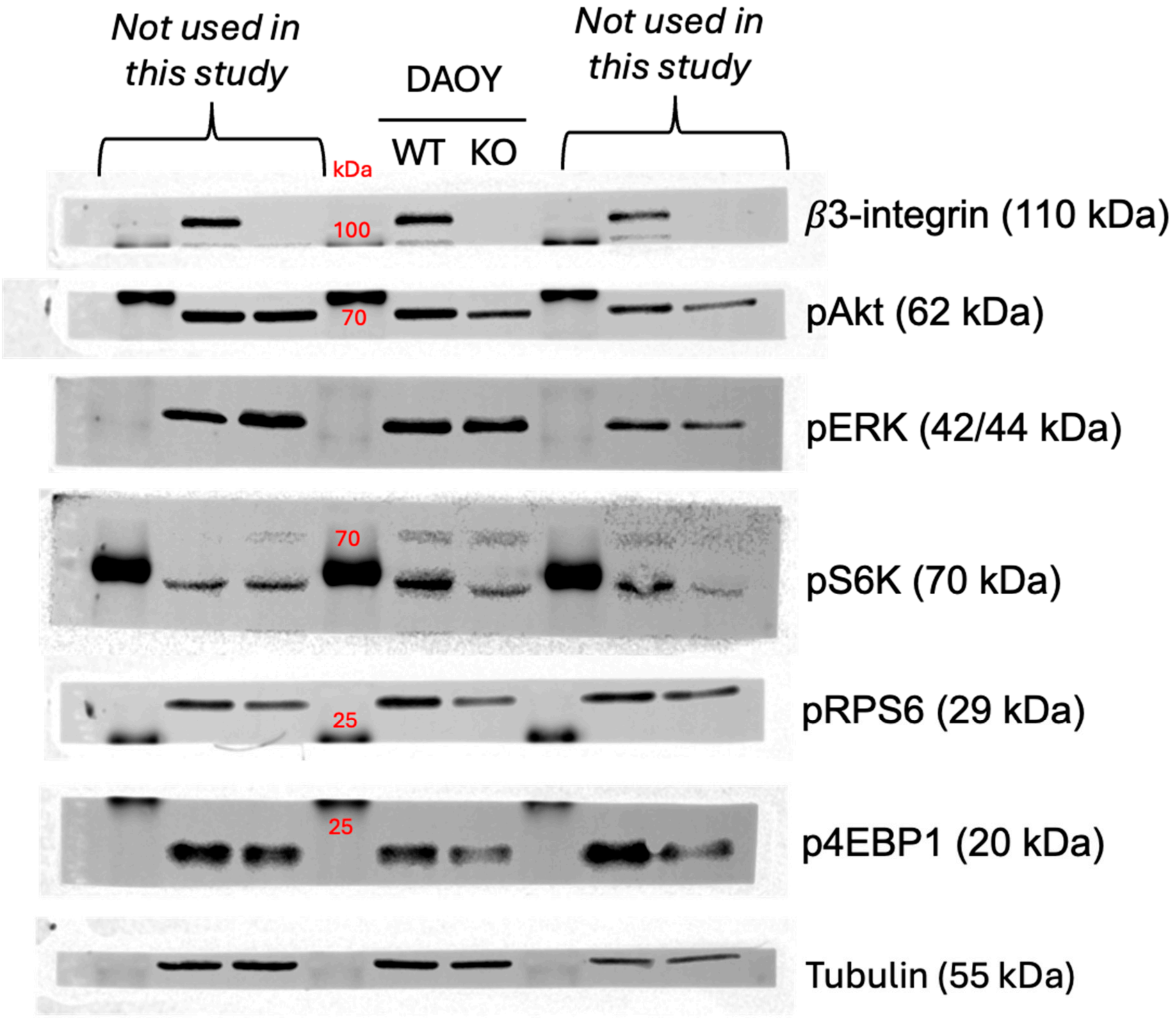

Figure 3B

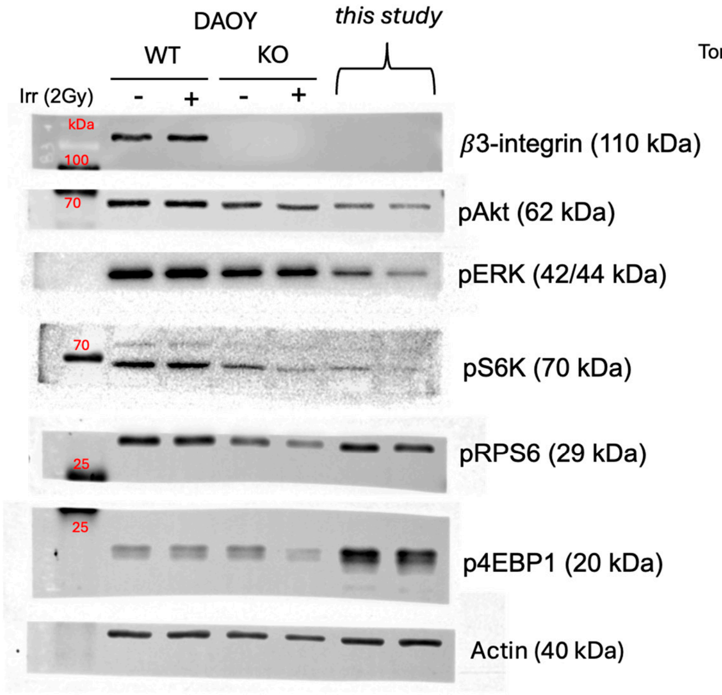

Figure 3C

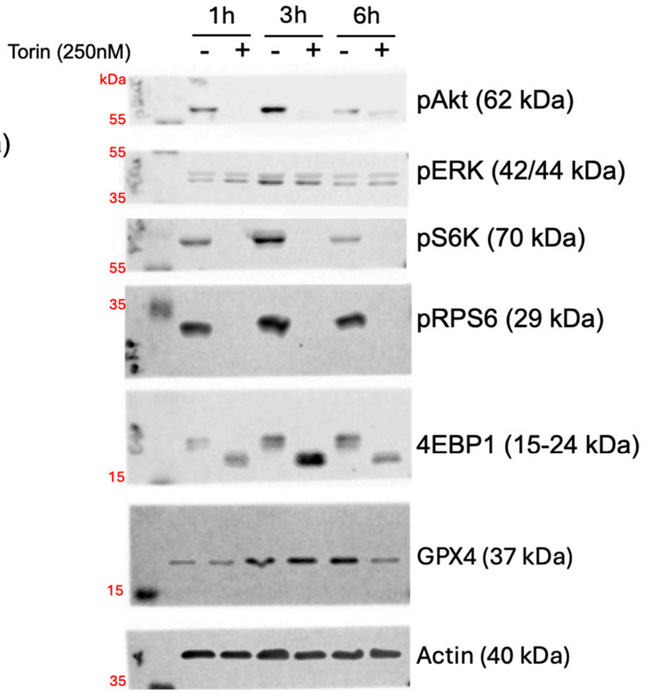

Figure S2

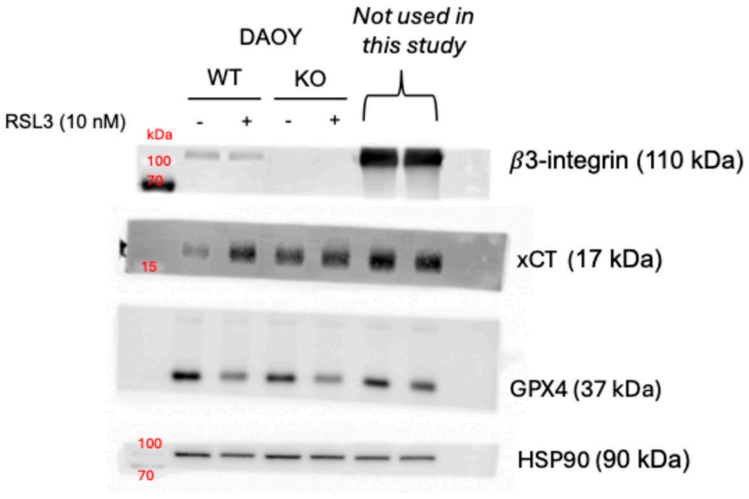

Figure S3

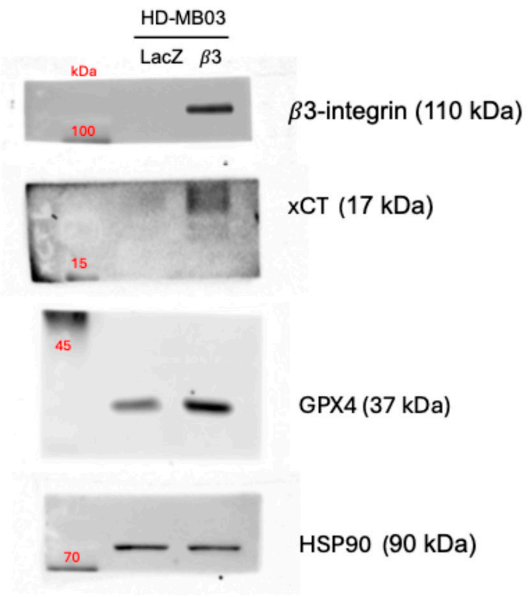

Figure S4

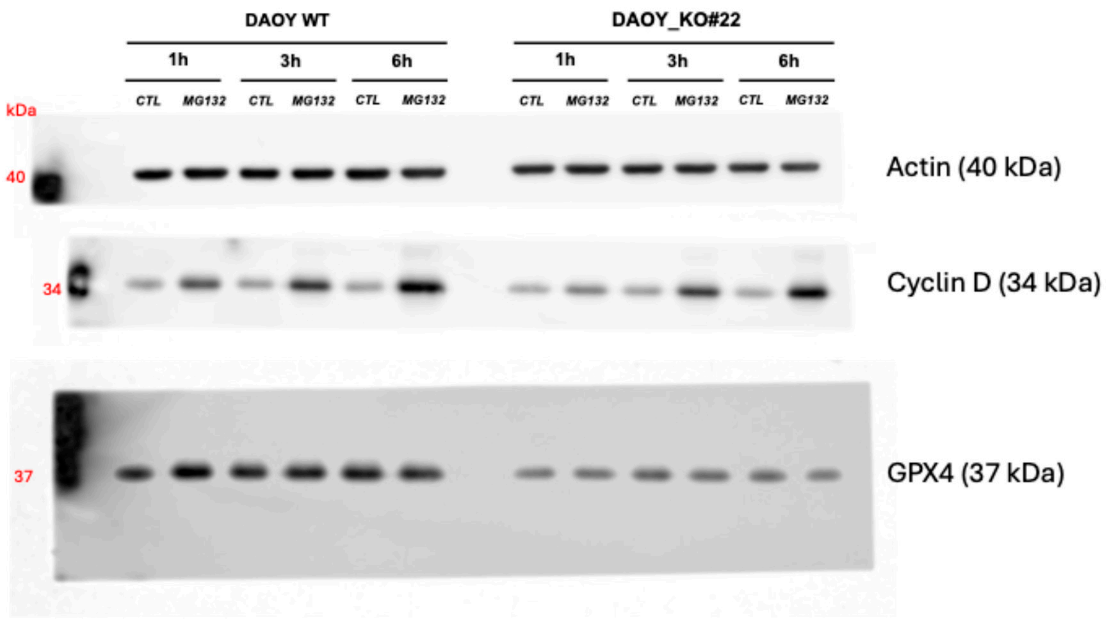

Figure S5

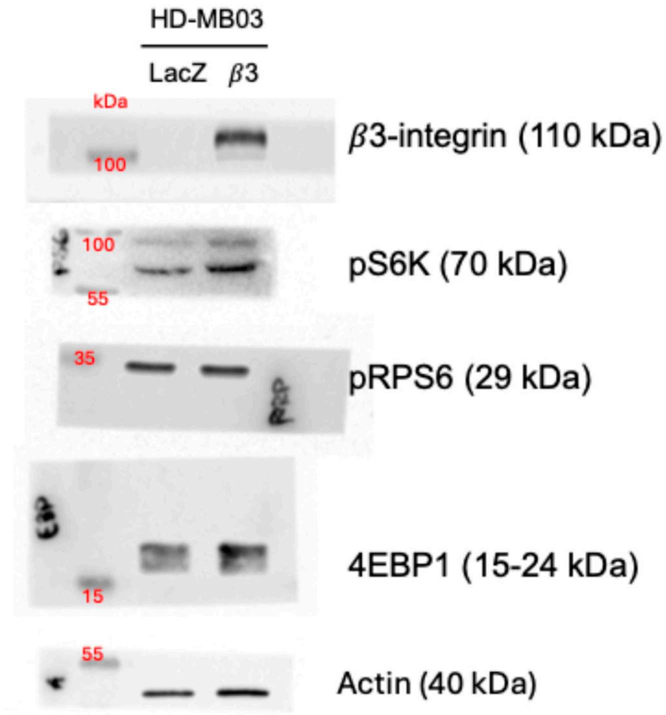

Figure S7

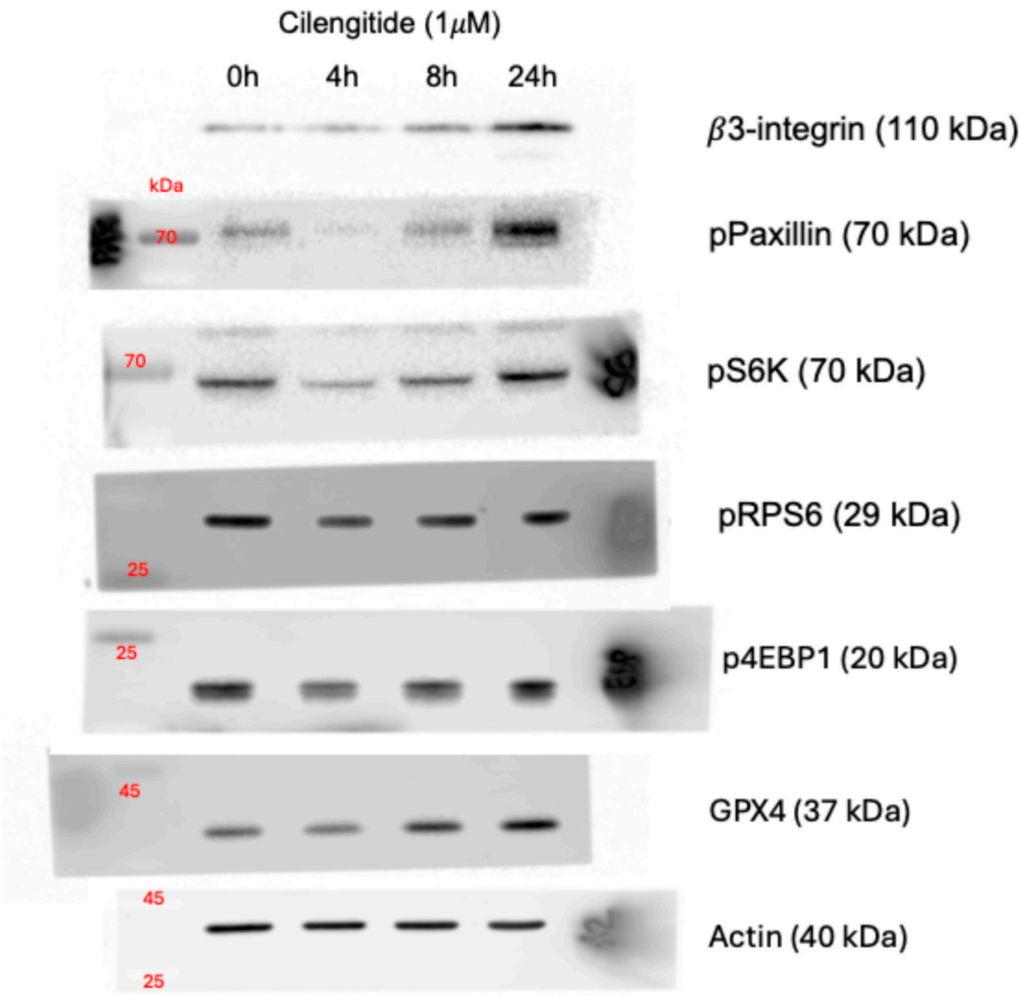

Cilengitide (1μM)
0h
4h
8h
24h
β3-integrin (110 kDa)
kDa
70
pPaxillin (70 kDa)
70
pS6K (70 kDa)
pRPS6 (29 kDa)
25
25
p4EBP1 (20 kDa)
45
GPX4 (37 kDa)
45
Actin (40 kDa)
25
